# The -2518 A/G polymorphism of the monocyte chemoattractant protein-1 as a candidate genetic predisposition factor for secondary myelofibrosis and biomarker of disease severity

**DOI:** 10.1038/s41375-018-0088-y

**Published:** 2018-03-06

**Authors:** Elena Masselli, Cecilia Carubbi, Benedetta Cambò, Giulia Pozzi, Giuliana Gobbi, Prisco Mirandola, Elena Follini, Luca Pagliaro, Daniela Di Marcantonio, Francesco Bonatti, Antonio Percesepe, Stephen M. Sykes, Franco Aversa, Marco Vitale

**Affiliations:** 1 Department of Medicine and Surgery, Unit of Biomedical, Biotechnological and Translational Sciences (S.Bi.Bi.T.), University of Parma, Parma, Italy; 2 Department of Medicine and Surgery, Unit of Clinical and Experimental Medicine, Hematology and BMT Unit, University of Parma, Parma, Italy; 3 Blood Cell Development and Function, Fox Chase Cancer Center, Philadelphia, PA USA; 4 Department of Medicine and Surgery, Unit of Medical Genetics, University Hospital of Parma, Parma, Italy; 5 CoreLab, University Hospital of Parma, Parma, Italy

Philadelphia-negative myeloproliferative neoplasms (MPN), namely polycythemia vera (PV), essential thrombocythemia (ET) and myelofibrosis (MF), are closely-related stem cell disorders, characterized by abnormal proliferation and differentiation of hematopoietic progenitors [1, 2]. Transitions between disease entities are common, shaping a “biological continuum” from an early stage with a relatively milder phenotype (PV and ET) toward an advanced phase, termed secondary myelofibrosis (sMF) [3]. Similarly, pre-fibrotic and overt primary myelofibrosis (pre-PMF and overt-PMF, respectively), according to the 2016 WHO criteria [4] have been shown to be aligned along a phenotypic gradient of severity [5]. Although different biomarkers have been associated with MPN thrombotic comorbidities [6, 7], no known parameters for predicting whether PV or ET will advance to sMF or for establishing a timeline for the progression of pre-PMF into overt disease currently exist.

Chronic inflammation plays a pivotal role in MPN pathogenesis, triggering neoplastic transformation and catalyzing clonal evolution toward end-stage disease. Indeed, MPN cells release a plethora of pro-inflammatory products, which in turn elicits genomic instability and drive clonal myeloproliferation [3, 8]. It has been demonstrated that: (i) MF patients display higher circulating levels of several pro-inflammatory cytokines as compared to other chronic myeloproliferative disorders as well as to healthy subjects [9], with IL-8, IL-2R, IL-12 and IL-15 levels independently holding prognostic value [10]; (ii) MCP-1 (monocyte chemoattractant protein-1, also known as CCL2), soluble IL-2R and IL-15 levels cluster with splenomegaly [11]; (iii) MCP-1 levels correlate with lower anemia response to pomalidomide [11].

MCP-1 is the main chemotactic factor for monocyte migration in sites of inflammation and contributes to organ fibrotic changes [12]. MCP-1 expression levels are highly variable among individuals, potentially contributing to differential susceptibility to various inflammatory conditions [13]. An A to G single-nucleotide polymorphism (SNP) in MCP-1 enhancer region (rs1024611, originally designated as –2518 G or –2578 G) was found to be responsible for higher levels of MCP-1 production by monocytes upon inflammatory noxa [14], and has been associated to several chronic inflammatory conditions such as autoimmune disorders, atherosclerosis and chronic infectious diseases [15]. In the present study, we investigated whether the -2518 A/G SNP of MCP-1 is a potential indicator of MPN susceptibility and/or disease phenotype.

After approval by the local ethical committee (prot. *n* 27182) and written informed consent, *n* 177 Caucasian MPN patients were recruited, of which *n* 44 PV, *n* 65 ET, *n* 68 MF (*n* 45 PMF and *n* 23 sMF). For PMF patients, histopathology, clinical and laboratory data were reviewed and diagnoses attributed to pre-PMF (*n* 12) or overt-PMF (*n* 33) according to the revised 2016 WHO criteria [4]. DNA was extracted by PureLink^®^ Genomic DNA Kit (Invitrogen) from 200 µl of whole blood and from buccal mucosa cells following manufacturer’s instructions. DNA from 149 age-matched and sex-matched Caucasian healthy subjects (CTRL) was provided by the Unit of Medical Genetics, University Hospital of Parma. Patients and CTRL genotyping was performed by TaqMan^®^ Predesigned SNP Genotyping Assays (Applied Biosystems). Patients’ data were retrospectively analyzed from cataloged hospital records.

For statistical analysis, numerical variables were summarized by their median and range, and categorical variables by count and relative frequency (percentage). Differences in the distribution of continuous variables were calculated by Mann–Whitney/Kruskal–Wallis tests, while categorical variable comparison were established by *χ*^2^/Fisher exact test. A *P* value <0.05 was considered statistically significant. Analysis was performed with dedicated software (Epi Info 7.2.1.0; CDC, Atlanta, GA, USA or StatView 5.0; SAS Institute Inc, Cary, NC, USA).

Case and control groups were aligned for age and gender distribution. Clinical and biological characteristics of MPN patients and CTRL are summarized in Supplemental Table 1.

Genotypic and allelic frequencies of the MCP-1 -2518 A/G SNP in MPN and CTRL are reported in Table 1. Genotypic frequencies were in Hardy–Weinberg equilibrium both in the MPN patients and CTRL (*P* > 0.05). No statistical differences were found by comparing genotypic and allelic frequencies of overall MPN, PV, ET and MF patients vs. CTRL, as well as between single disease entities.

**Table 1:** Genotypic and allelic frequencies of the -2518 A/G SNP of MCP-1 in overall MPN population, PV, ET, MF patients and CTRL

	Genotypic frequencies				Allelic frequencies	
	A/A *n* (%)	A/G *n* (%)	G/G *n* (%)	A/G + G/G *n* (%)	A allele	G allele
MPN (*n* 177)	94 (53.1)	74 (41.8)	9 (5.1)	83 (46.9)	0.740	0.260
PV (*n* 44)	26 (59.1)	15 (34.1)	3 (6.8)	18 (40.9)	0.761	0.239
ET (*n* 65)	31 (47.7)	33 (50.8)	1 (1.5)	34 (52.3)	0.731	0.269
MF (*n* 68)	37 (54.4)	26 (38.2)	5 (7.4)	31 (45.6)	0.735	0.257
CTRL (*n* 149)	90 (60.4)	53 (35.6)	6 (4.0)	59 (39.6)	0.782	0.218

*P* n.s. in all comparisons

Focusing on MF, which is the MPN variant characterized by the highest inflammation burden [16], we evaluated whether polymorphic genotypes could be associated to specific disease subtype(s) (based on the 2016 WHO criteria) or to disease phenotype aggressiveness based on the hematologic characteristics at the time of diagnosis (Table 2).

**Table 2:** Genotype–phenotype correlations in MF patients

	No. of cases	A/A	A/G + G/G	*P* [O.R., 95% C.I.]
Disease type				
PMF, *n* (%)	45	31 (68.9)	14 (31.1)	***P*** = **0.0008 vs. sMF** **[6.23; 2.04–19.32]**
Pre-PMF, *n* (%)	12	11 (91.7)	1 (8.3)	***P*** = **0.0002 vs. sMF** **[31.17; 3.29–295.35]** *P* = 0.07 vs. overtPMF
Overt-PMF, *n* (%)	33	20 (60.6)	13 (39.4)	***P*** = **0.011 vs. sMF** **[4.36; 1.36–13.95]**
sMF *n* (%)	23	6 (26.1)	17 (73.9)	***P*** = **0.022 vs. CTRL** **[3.07; 1.14–8.32]**
Age				
Median (range), years	68	69.0 (29–84)	70.0 (30–86)	*P* = 0.61
>65 years, *n* (%)	46	25 (54.4)	21 (45.6)	*P* = 0.99
Gender				
Male, *n* (%)	41	21 (51.2)	20 (48.8)	*P* = 0.51
Female, *n* (%)	27	16 (59.3)	11 (40.7)	
IPSS				
Low/intermediate-1, *n* (%)	42	28 (66.7)	14 (33.3)	***P*** = **0.0078** **[4.29; 1.42–12.91]**
Intermediate-2/high, *n* (%)	22	7 (31.8)	15 (68.2)	
Hemoglobin				
Median (range), g/L	62	12.7 (5–15.9)	11.7 (7.3–15.5)	*P* = 0.062
<100 g/L, *n* (%)	13	4 (30.8)	9 (69.2)	***P*** = **0.036** **[3.89; 1.04–14.41]**
WBC				
Median (range), x10^9^/L	62	9.2 (3.9–57.8)	12.1 (2.9–57.0)	*P* = 0.78
<4 × 10^9^/ L or >25 × 10^9^/L, *n* (%)	7	3 (42.9)	4 (57.1%)	*P* = 0.44
Platelets				
Median (range), ×10^9^/L	60	560 (99–1322)	376 (69–984)	*P* = 0.10
LDH				
Median (range), U/L	59	622 (205–1620)	751 (343–1580)	*P* = 0.22
>Normal range, *n* (%)	47	26 (55.3)	21 (44.7)	*P* = 0.85
Constitutional symptoms				
Yes, *n* (%)	47	7 (41.8)	10 (58.8)	*P* = 0.19
No, *n* (%)	17	28 (59.6)	19 (40.4)	
Circulating blasts				
<1%, *n* (%)	53	33 (62.3)	20 (37.4)	***P*** = **0.014** **[6.6; 1.27–34.23]**
≥1%, *n* (%)	10	2 (20.0)	8 (80.0)	
Grading of fibrosis				
0–I, *n* (%)	29	20 (69.0)	9 (31.0)	***P*** = **0.048** **[2.78; 0.99–7.43]**
≥II, *n* (%)	36	16 (44.4)	20 (55.6)	
Spleen (long. Ø by US) median (range), cm	68	14.0 (7.5–30)	17.0 (10–30)	*P* = 0.1
*JAK2*V617F mutation				
Positive, *n* (%)	40	21 (52.5)	19 (47.5)	*P* = 0.44
Negative, *n* (%)	19	12 (63.2)	7 (36.8)	
Major thrombotic events				
Yes, *n* (%)	22	11 (50.0)	11 (50.0)	*P* = 0.55
No, *n* (%)	45	26 (57.8)	19 (42.2)	

Statistically significant associations are highlighted in bold, and relative Odds ratio (O.R.) and 95% Confidence Interval (C.I.) are reported

Age, IPSS risk category, leukocytes, hemoglobin, platelets, presence of blasts, LDH constitutional symptoms and spleen size refer to the time of diagnosis. “No. of cases” (second column) refers to: (i) for non-continuous variables, the no. of patients presenting the indicated parameter (i.e., no. of JAK2V617 positive and negative patients); (ii) for continuous variables, the no. of patients evaluated for that parameter (i.e., age at the time of diagnosis).

We found that the subjects carrying either a heterozygous or homozygous genotype for the -2518 A/G SNP (A/G + G/G) were significantly more frequent in sMF vs. PMF (17/23, 73.9% vs. 14/45, 31.1%, respectively, *P* = 0.0008, Table 2). Additionally, sMF was significantly more frequent in A/G + G/G patients than either pre-PMF (1/12, 8.3%, *P* = 0.0002) or overt-PMF (13/33, 39.4%, *P* = 0.011). Notably, the number of A/G + G/G subjects was also significantly higher in sMF as compared to CTRL (*P* = 0.022) (Table 2). The observation that sMF is enriched in allele-G carriers is consistent with the concept of myelofibrosis as a burn-out phase of a long process that starts with ET/PV and advances toward a more progressive disease state, characterized by higher inflammation burden [3, 16]

Genotype–phenotype correlation studies in MF patients revealed a higher frequency of allele-G carriers (A/G + G/G) in: (i) intermediate-2/high vs. low/intermediate-1 IPSS risk group (15/22, 68.2% vs. 14/42, 33.3%, respectively, *P* = 0.0078), (ii) patients with lower (Hb < 100 g/L) vs. higher (Hb ≥ 100 g/dL) hemoglobin levels (9/13, 69.2% vs. 18/49, 36.7%, *P* = 0.036, (iii) patients with ( ≥ 1%) vs. patients without (<1%) circulating blasts (8/10, 80%, vs. 20/53, 37.4%, *P* = 0.014), (iv) patients with higher (≥II) vs. lower (0–I) grading of bone marrow fibrosis (20/36, 55.6% vs. 9/29, 31.0%, *P* = 0.048) (Table 2).

No associations with age, gender, white blood cell and platelet count, LDH levels, presence of constitutional symptoms, spleen size, JAK2V617F mutation, and history of major thrombotic events were found (Table 2).

Finally, to evaluate whether the MCP-1 -2518 A/G SNP is inherited or acquired by hematopoietic stem cells, we tested the SNP in non-clonal cells of 14 MPN patients (10 MF, 3 ET and 1 PV) harboring the G allele, as assessed by whole blood genotyping. The analysis of buccal mucosal cells revealed that all individuals were germline carriers of the polymorphism.

In conclusion, our data suggest that the -2518 A/G SNP of MCP-1 could represent a host genetic predisposition factor for sMF and may serve as a biomarker of disease severity in MF, as implied by its association with higher IPSS, peripheral blasts, lower hemoglobin and higher grading of bone marrow fibrosis. In particular, the association of the SNP with higher grading of bone marrow fibrosis as well as with severe anemia is consistent with the well-defined pro-fibrotic role of this chemokine [12] and the previously described observation that MCP-1 levels correlates with poor anemia response [11]. We speculate that this SNP, after prospective validation studies, may configure as a genetic biomarker identifying ET and PV patients who more likely will progress toward a spent phase.

## Electronic supplementary material

Supplemental Table 1(DOCX 19 kb)
