# Supplementary material for: The -2518 A/G polymorphism of the monocyte chemoattractant protein-1 as a candidate genetic predisposition factor for secondary myelofibrosis and biomarker of disease severity
Source: Leukemia. 2018 Mar 6;32(10):2266–70. doi: 10.1038/s41375-018-0088-y (PMC6170394; doi:10.1038/s41375-018-0088-y)
Supplement: Supplementary file 1 — Supplemental Table 1(DOCX 19 kb) [file 41375_2018_88_MOESM1_ESM.docx]

|  | PV  (tot. n. 44) | ET  (tot. n. 65) | MF  (tot. n. 68) | CTRL  (tot. n. 149) |
| --- | --- | --- | --- | --- |
| **Age (diagnosis)**  Median (range), y | 67.0 (26-86) | 52.8 (11-85) | 70.0 (29-86) | 63.0 (29-85) |
| **Male pts**  n./tot. (%) | 26/44 (59.0) | 33/65 (50.8) | 41/68 (60.3) | 83/149 (55.7) |
| **Follow-up**  Median (range), y | 4.0 (0-22) | 6 (0-26) | 3.0 (0-22) | *N/A* |
|  |  |  | 6.5 (0-38)* |  |
| ***JAK2*V617F**  Mutated, n./tot. tested (%) | 42/44 (95.5) | 31/55 (56.4) | 40/59 (67.8) |  |
| ***MPL*W515**  Mutated, n./tot. tested (%) | *N/E* | 1/16 (6.3) | 1/12 (8.3) |  |
| ***CALR* (type 1&2)**  Mutated, n./tot. tested (%) | *N/E* | 7/16 (43.8) | 8/11 (72.7) |  |
| **Pts with major thrombotic events^#^**  n./tot. evaluated (%) | 8/42 (19.1) | 13/57 (22.8) | 22/67 (32.8) |  |

**Supplemental Table 1. Clinical and biological characteristics of PV, ET, MF patients and control subjects (CTRL)**

**Including previous PV/ET*

*#Major thrombotic events included: acute myocardial infarction; TIA/stroke, peripheral arterial thrombosis, venous thrombosis embolism as defined by Barbui T. et al. 2016^6^.*

*N/E: not evaluated. N/A: not applicable*

*P= n.s. by comparing age and gender distribution among all groups*
